# Comparison of Methods to Reduce Myocardial 18F-FDG Uptake in Mice: Calcium Channel Blockers versus High-Fat Diets

**DOI:** 10.1371/journal.pone.0107999

**Published:** 2014-09-19

**Authors:** Lorena Cussó, Juan José Vaquero, Stephen Bacharach, Manuel Desco

**Affiliations:** 1 Departamento de Bioingeniería e Ingeniería Aeroespacial, Universidad Carlos III de Madrid, Madrid, Spain; 2 Instituto de Investigación Sanitaria Gregorio Marañón, Madrid, Spain; 3 Department of Radiology, Nuclear Medicine Section, University of California San Francisco, San Francisco, California, United States of America; 4 Centro de Investigación Biomédica en Red de Salud Mental (CIBERSAM), Madrid, Spain; University of Alabama at Birmingham, United States of America

## Abstract

**Purpose:**

Besides its application in oncology, ^18^F-FDG PET-CT imaging is also useful in the diagnosis of certain lung infections, inflammatory diseases, and atherosclerotic plaques. Myocardial uptake of ^18^F-FDG may hamper visualization of the lesions caused by these diseases. Two approaches have been proposed for reducing myocardial uptake in preclinical studies, namely, calcium channel blockers (verapamil) and high-fat diets such as commercial ketogenic diets and sunflower seed diets. The objective of this study was to compare the efficacy of these approaches in reducing myocardial uptake of ^18^F-FDG in mice.

**Methods:**

We performed two experiments. In experiment A, each animal underwent four ^18^F-FDG PET/CT scans in the following order: baseline, after administration of verapamil, after two days on ketogenic diet and after two days on sunflower seeds. PET scans were performed 60 minutes after injection of 18.5 MBq of ^18^F-FDG. In experiment B, the best protocol of the three (ketogenic diet) was evaluated in a lung inflammation model to assess the efficacy of reducing myocardial uptake of ^18^F-FDG.

**Results:**

Compared with baseline (SUV 2.03±1.21); the greatest reduction in uptake of ^18^F-FDG was with ketogenic diet (SUV 0.79±0.16; p = 0.008), followed by sunflower seeds (SUV 0.91±0.13; p = 0.015); the reduction in myocardial uptake produced by verapamil was not statistically significant (SUV 1.78±0.79; p = NS). In experiment B, complete suppression of myocardial uptake noticeably improved the visualization of inflamed areas near the heart, while in the case of null or partial myocardial suppression, it was much harder to distinguish lung inflammation from myocardial spillover.

**Conclusion:**

A high-fat diet appeared to be the most effective method for decreasing myocardial uptake of ^18^F-FDG in healthy mice, outperforming verapamil. Our findings also demonstrate that ketogenic diet actually improves visualization of inflammatory lesions near the heart.

## Introduction

Positron emission tomography (PET) with 2-deoxy-2-(^18^F)fluoro-D-glucose (^18^F-FDG) is an essential tool in oncology imaging [1]. Given that both tumor and immune cells have high glucose metabolism, the role of ^18^F-FDG-PET/computed tomography (CT) is being expanded in clinical practice for the detection of inflammatory and infectious diseases. However, some organs, such as the heart, brain, and liver, also have high glucose metabolism and, consequently, show high ^18^F-FDG uptake in the absence of disease. Interference from myocardial uptake may hamper visualization of lesions – and thus diagnosis – in several diseases, such as visualization of carotid plaques or pulmonary or cardiac inflammatory/infectious (e.g., endocarditis) [2] and lung infections (e.g., tuberculosis). Minimizing myocardial uptake of ^18^F-FDG can improve detectability of lesions in the middle pulmonary lobe and adjacent mediastinal tissues and decrease the number of false-positive readings [3], [4], [5]. The two main mechanisms for reducing myocardial uptake of ^18^F-FDG are switching myocardial metabolism to free fatty acids (FFAs) and blocking glucose transport into cardiomyocytes.

The first mechanism takes advantage of the ability of the myocardium to switch its metabolism from glucose to FFAs in order to reduce myocardial ^18^F-FDG needs. This is possible because cardiomyocytes can use a variety of energy sources, such as FFAs, glucose, lactate, and ketone bodies.

In fasting states, or under high-fat diets, heart energy production is primarily based on FFAs (Randle cycle) rather than on glucose metabolism. Conversely, the ingestion of carbohydrates or glucose raises plasma concentrations of insulin and glucose and reduces the availability of FFAs in the myocardium [2], [6]. Alternatively, a pharmacological approach could facilitate patient cooperation; heparin, for instance, is known to increase the concentration of FFAs in blood [7].

Nowadays, clinical protocols to reduce myocardial uptake of ^18^F-FDG are moving from fasting-based methods to dietary protocols (low-carbohydrate or low-carbohydrate plus high-fat diets), because a substantial reduction in myocardial uptake requires long fasting periods that make patient cooperation problematic [8]. On the other hand, one night on a low-carbohydrate, high-fat diet seems sufficient to ensure good myocardial suppression [9]. Despite efforts to standardize patient preparation before PET studies, myocardial uptake of ^18^F-FDG has proven highly variable in both clinical and preclinical applications owing to individual patient characteristics [10], [11] or to differences in animal handling [12], [13].

Blocking glucose transport into cardiomyocytes can also use to suppress myocardial uptake of ^18^F-FDG. Verapamil is a calcium channel antagonist that modulates the influx of calcium across the membrane of the arterial smooth muscle cells and cardiomyocytes. It is used to treat hypertension, angina pectoris, and cardiac arrhythmia. Verapamil reduces myocardial glucose uptake by blocking calcium channels. In a preclinical study, administration of verapamil before ^18^F-FDG led to a significant reduction in myocardial uptake in healthy mice [14].

Both approaches – high-fat diets and verapamil – are thought to considerably reduce myocardial uptake of ^18^F-FDG; however, to our knowledge, no comparative studies have been performed. The primary objective of our work was to assess (both quantitatively and visually) and compare the efficacy of high-fat diets and verapamil to suppress myocardial uptake of ^18^F-FDG in healthy mice. Our secondary objective was to evaluate the usefulness of the method for improving the detectability of lesions in a murine lung inflammation model.

## Material and Methods

### Ethics statement

All animal procedures were approved by the Animal Experimentation Ethics Committee of Hospital General Universitario Gregorio Marañón, Madrid, Spain and were performed according to EU directive 2010/63/EU and national regulations (RD 53/2013).

### Experimental procedures

**Figure 1:**
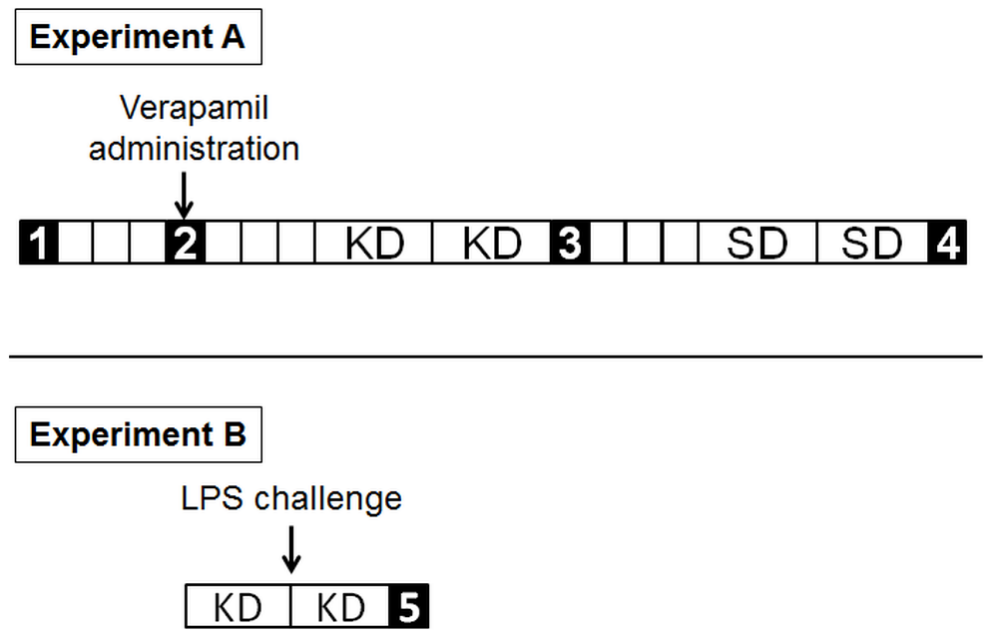
Experimental workflow. Each box represents a day. The numbers indicate the ^18^F-FDG PET/CT scans. Experiment A: (1) baseline, (2) post-verapamil, (3) post-ketogenic diet (KD) and (4) post-sunflower seed diet (SD). Experiment B: LPS challenge was performed 24 hours before the PET scan (5).

Ten female C57BL/6 mice aged eight weeks (Harlan Laboratories) were housed in cages under standard environmental conditions and allowed food and water ad libitum. As the experimental workflow shows (Fig. 1), two experiments were carried out in this study. In experiment A, each animal underwent four ^18^F-FDG PET/CT scans as follows: baseline, after administration of verapamil, after two days on a ketogenic diet, and after two days on a sunflower seed diet. Mice returned to their regular diet (A04, SAFE) for four days between each treatment or change in diet.

Once experiment A was completed, the resulting best protocol was evaluated in a lung inflammation model (n = 10) to assess the efficacy of reducing myocardial uptake of ^18^F-FDG (Fig. 1 Experiment B).

#### Verapamil

Mice received a single intraperitoneal dose of 1.3 mg/kg (Manidón 2.5 mg/2 ml) one hour before administration of ^18^F-FDG [14].

#### Diets

Two high-fat diets were tested in this study: a commercial ketogenic diet (TD 96355, Harlan) and a sunflower seed diet (Euricar Europa, S.L.). The ketogenic diet is very high-fat (90%/0.4%, fat/carbohydrates) with almost no carbohydrate, while sunflower seeds are an intermediate fat diet (55%/18%, fat/carbohydrates) [15].

#### Lung inflammation model

We administered 5 mg/kg of lipopolysaccharide (LPS) from *Escherichia coli* (055:B5 Sigma-Aldrich) diluted in 100 µl of saline into the mouse trachea under light anesthesia with isoflurane [16]. The ^18^F-FDG PET scan was performed 24 hours after the LPS challenge (Fig. 1 Experiment B).

#### Histopathology

Forty-eight hours after the LPS challenge and after the last PET study, the animals were euthanized using a CO_2_ chamber. The lungs were fixed in 10% neutral buffered formalin. Sections (5 µm) of the left and right lung lobes were placed into a tissue-processing cassette and stained with hematoxylin and eosin.

### PET/CT imaging

All the studies were performed with a small-animal PET/CT device (Argus, SEDECAL, Spain [17], [18]). PET scans were acquired 60 minutes after intraperitoneal administration of 19.4±1.02 MBq of ^18^F-FDG. During uptake, the animals were kept awake and warm under infrared light to avoid brown fat uptake. Blood glucose levels (BGLs) were measured before administration of ^18^F-FDG using a glucose meter (Glucocard TM G+ meter, A. Menarini, Spain). PET data were collected for 45 minutes with the mice anaesthetized using 1.5% isoflurane in oxygen at 3 l/min and reconstructed using OSEM-2D with 50 subsets and 2 iterations. The voxel size of the reconstructed images was 0.388 mm in the transaxial plane and 0.775 mm axially. After the PET scan, a CT study was acquired using an X-ray beam current of 240 µA and a tube voltage of 40 kVp and reconstructed using an FDK algorithm [19]. These CT scans were used as anatomical templates and to perform intra-subject spatial co-registration.

### Data analysis

#### Visual uptake scale

**Figure 2:**
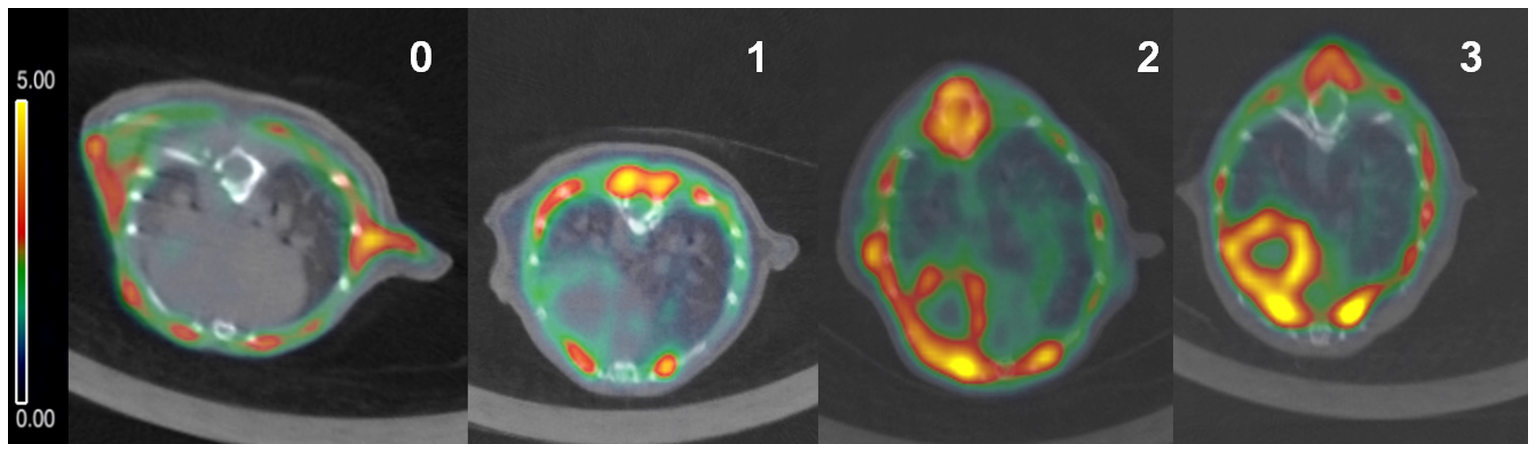
Four-grade visual uptake scale. 0, homogeneously minimal; 1 mostly minimal or mild; 2 mostly intense or moderate; 3 homogeneously intense.

Five experts rated in a blinded fashion the ^18^F-FDG myocardial uptake according to the Likert scale [5], as follows: 0, homogeneously minimal; 1 mostly minimal or mild; 2 mostly intense or moderate; 3 homogeneously intense (Fig. 2).

#### Standardized uptake value (SUV)

For each image, a region of interest (ROI) was selected containing the myocardium of the whole left ventricle (8–10 slices). The mean myocardial SUV was calculated in each subject. In order to use exactly the same ROIs, studies of suppressed uptake were manually co-registered to the baseline PET scan using the CT scan of the thorax.

#### Lesion/myocardium ratio

For each animal on Experiment B, a lesion and heart ROI were selected on a slice from the CT study containing both lesion and heart areas. These ROIs were applied to the co-registered PET images in order to measure the maximum activity within each ROI and calculate the lesion/myocardium ratio.

#### Statistical analysis

Agreement of the visual assessment between experts was measured using the Cohen's kappa coefficient. Differences between treatments were compared using a Wilcoxon test. Data are reported as mean (± standard deviation). Differences in BGLs and myocardial uptake were compared between the experimental treatments using repeated-measurements one-way ANOVA with Sidak post hoc test. Data were logarithmically transformed because they did not follow a Gaussian distribution (Kolmogorov-Smirnov, p = 0.028 on original myocardial data and p = 0.256 after log-transformation). The differences between the lesion/myocardium ratios were compared using one-way ANOVA with Sidak post hoc test. Differences were considered statistically significant at p<0.05.

## Results

### Experiment A

Animals in experiment A were divided into four groups based on uptake of ^18^F-FDG, measured according to Likert scale (Fig. 2). Kappa coefficient showed a substantial agreement between experts (0.6366), according to standard kappa interpretation [20]. This assessment of the PET images for each treatment is shown in Table 1. After administration of verapamil, no animal showed complete myocardial suppression (0 on the Likert scale), whereas it was homogeneously intense in 40% of the animals. Sunflower seeds suppressed myocardial uptake in 40% of the animals, while the ketogenic diet suppressed uptake in 70%. Uptake was not intense in animals on the ketogenic diet or in animals fed sunflower seeds. Wilcoxon tests showed significant differences between verapamil and seed diet (p = 0.013) and between ketogenic diet and seed diet (p = 0.037).

**Table 1:** Image assessment of myocardial uptake of ^18^F-FDG.

Likert scale	Post-Verapamil	Post-Seeds	Post-KD
**0** (homogeneously minimal)	0	4	7
**1** (mostly minimal)	2	4	3
**2** (mostly intense)	4	2	0
**3** (homogeneously intense)	4	0	0

After each treatment animals were divided into four groups based on visual myocardial ^18^F-FDG uptake (see Fig. 2).

**Figure 3:**
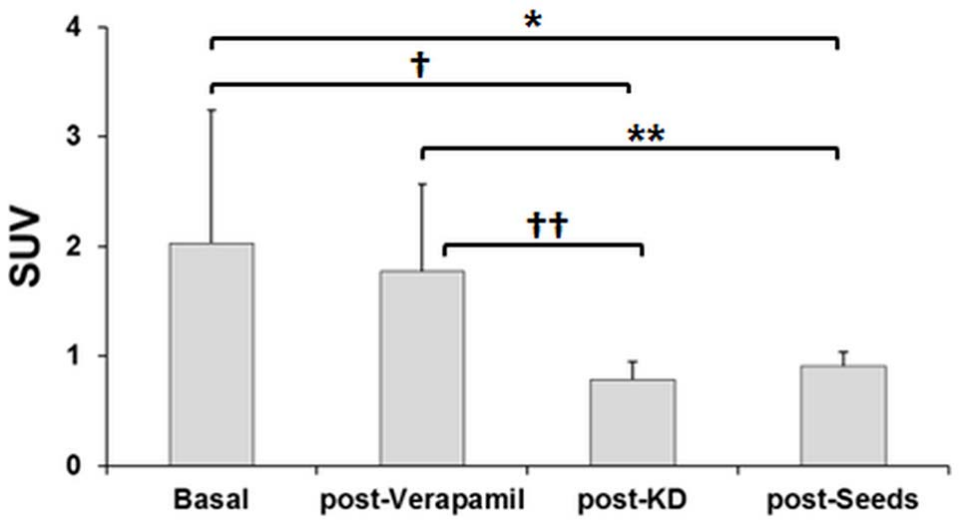
Plots of standardized uptake values mean (SUV) of left ventricular myocardium after each treatment. p values were calculated using the Sidak post hoc test (*p = 0.015, †p = 0.008, **p = 0.007and ††p = 0.003).

Quantification of myocardial SUV (Fig. 3) showed that, compared with baseline (SUV 2.03±1.21), the greatest reduction in ^18^F-FDG uptake was with a ketogenic diet (SUV 0.79±0.16; p = 0.008), followed by sunflower seeds (SUV 0.91±0.13; p = 0.015); verapamil produced no significant reduction in myocardial uptake (SUV 1.78±0.79; p = NS). The differences in uptake observed between the two diets did not reach statistical significance (p = 0.626), p values were obtained using the Sidak post hoc test. SUV results combined with the visual assessment further support that ketogenic diet led to the greatest reduction in myocardial uptake, followed by sunflower seeds.

Table 2 shows BGLs before administration of ^18^F-FDG for each treatment. The ketogenic diet significantly decreased blood glucose concentration compared with baseline and verapamil (p = 0.04 and p = 0.004, respectively, Sidak post hoc tests).

**Table 2:** Blood glucose level (BGL) before ^18^F-FDG administration.

Treatment	BGL, mg/dl
Baseline	110.7±21.3
Post-Verapamil	121.5±16.9
Post-KD	75.7±25.7*†
Post-seeds	91.1±33.0

Mean BGL (± SD). p values were calculated using the Sidak post hoc test (*p = 0.04 vs. baseline; †p = 0.004 vs. verapamil).

### Experiment B

**Figure 4:**
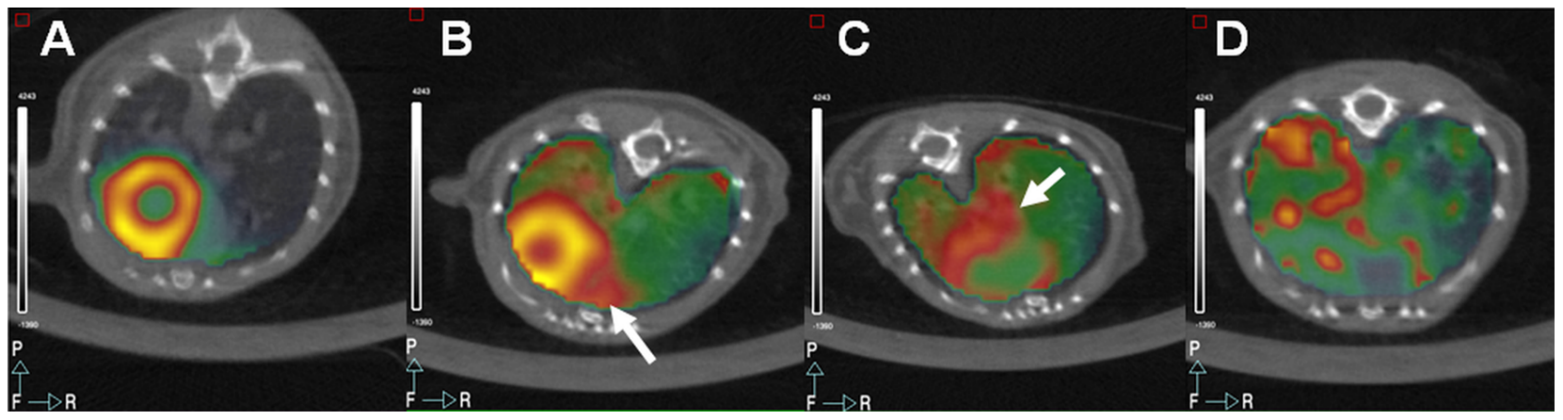
Lung inflammation visualized by ^18^F-FDG-PET. (A) Control PET scan after regular diet in a non–LPS-challenged animal. Lungs do not show ^18^F-FDG uptake. (B–D) LPS-challenged animals after two days of ketogenic diet. (B) Null suppression of myocardial uptake. (C) Partial suppression of myocardial uptake. In the case of null or partial suppression, it is much harder to distinguish inflammation from myocardial spillover (arrows). (D) Complete suppression improves hot areas identification.

After the LPS challenge, all the animals (n = 10) developed severe respiratory problems, and two died within the first 30 hours. Positive uptake of ^18^F-FDG in lung tissue was recorded in all animals 24 hours after LPS compared with baseline (Fig. 4A). Animals in experiment B were also divided into four groups based on the Likert scale, as described above (Fig. 2). Myocardial uptake was completely suppressed in 50% of the animals, 30% showed a moderate reduction, and 20% had homogeneously intense uptake. Figure 4 shows an example of lung inflammation according to the degree of myocardial uptake of ^18^F-FDG. As expected, complete suppression of myocardial uptake noticeably improved the visualization of hot areas near the heart (Fig. 4D), whereas in the case of null or partial suppression, it was much harder to distinguish inflammation from myocardial spillover (arrows on Fig. 4B and 4C). Lesion/myocardium ratio was higher in animals with complete myocardium suppression (1.4±0.2) compared with moderate myocardium reduction (0.71±0.11; p = 0.013) and also with null reduction (0.8±0.03; p = 0.006). Lung histology confirmed the existence of inflammatory lesions (data not shown).

## Discussion

The aim of this study was to assess the efficacy of high-fat diets and administration of verapamil for suppression of myocardial uptake of ^18^F-FDG in mice. Our results showed that, when given over two days, both ketogenic and sunflower diets reduced myocardial uptake of ^18^F-FDG significantly more than administration of verapamil. This finding held true both visually (Table 1) and quantitatively (Fig. 3).

Studies in humans have shown that diet protocols can suppress myocardial uptake of ^18^F-FDG better than fasting methods [21]. A study by Kobayashi et al. [8] reported complete suppression in 71.4% of healthy volunteers after 24 hours of carbohydrate restriction combined with a high-fat drink one hour before ^18^F-FDG, while the remaining 28.6% showed only residual uptake of ^18^F-FDG by the papillary muscle. Williams and Kolodny [5] reported a larger reduction in myocardial SUV in cancer patients with a low-carbohydrate/high-fat dietary protocol three to six hours before ^18^F-FDG than in patients following a fasting protocol (3.9±3.6 and 8.8±5.7). Furthermore, Cheng et al. [22] reported that a low carbohydrate diet suppressed myocardial uptake better than a standard fasting protocol and that a low-carbohydrate protocol was better than a low-carbohydrate/high-fat protocol in cancer patients. The myocardial uptake values in the control group (fasting group) reported by Cheng et al. [22] were lower than those in Kobayashi et al. [8] and Williams et al. [5]. The authors attributed this difference to variability in myocardial uptake [22]. Khandani et al. [11] examined myocardial variability and showed that cardiac ^18^F-FDG uptake did not change on serial scans of the same patient over time, but did change when groups of patients were compared. This variability in myocardial uptake between patients may make it difficult to standardize their preparation before a PET scan.

Despite the controversy surrounding reduction in myocardial uptake in human studies, the present findings are consistent with those of previous preclinical works. Tupper et al. [23] showed reduced cardiac uptake in mice after 12 hours of ketogenic diet compared with 12 hours of fasting. Fine et al. [24] compared the effects on myocardial uptake of three diets in rats (low carbohydrate/high-fat, 0.01%/90%; intermediate, 52%/28%; and high carbohydrate/low fat, 78%/2%) and showed that the low carbohydrate/high-fat diet induced a 70% reduction in myocardial uptake. In the present study, two days on ketogenic diet was enough to induce a 61% reduction in ^18^F-FDG uptake compared with baseline, while a sunflower seed diet reduced uptake by about 55%, perhaps because the latter has a lower content of FFAs than the ketogenic diet. Nevertheless, cardiac ^18^F-FDG can be somewhat reduced using a sunflower seed diet; in addition, its low cost makes it a very promising option. We hypothesize that increasing the duration of the diet by one or two days could be enough to produce effects similar to those reported by Fine et al. [24]. No optimal protocols for the duration of diets have been reported to reduce myocardial ^18^F-FDG uptake. In recent clinical studies, duration of diet varies between one night [9] and 24 hours [8]. In preclinical settings, durations of 12 hours [23] to 4 weeks [24] have been reported. Although one night seems to be sufficient, diet protocols nowadays involve two or three fat meals (24 hours) before the PET scan; in the case of rodents, this could be equivalent to two nights. Our results confirm that two days of a high-fat diet is sufficient to suppress myocardial ^18^F-FDG uptake.

Diets have proven successful in mice; however, in humans, they require good adherence, which is not always easy to achieve. Wykrzykowska et al. [9] showed inadequate cardiac suppression in 12 of 32 patients who admitted to not adhering to their diet. Therefore, pharmacological approaches are often used to avoid non-adherence. Ishimura et al. [7] administered heparin before ^18^F-FDG to detect sarcoidosis lesions, because heparin increases serum FFA levels and possibly minimizes background myocardial uptake. Gaeta et al. [14] used a calcium channel blocker (verapamil) to avoid glucose transport into cardiomyocytes and thus reduce ^18^F-FDG uptake in animals. Our findings showed that after administering verapamil one hour before injection of ^18^F-FDG, only one out of ten animals exhibited minimal myocardial uptake (0 on the Likert scale) and only two of ten obtained mostly minimal uptake (1 on the Likert scale). However, consistent with Gaeta et al. [14], we observed a slight (12.7%) reduction in myocardial uptake with verapamil (Fig. 3) compared to baseline, but the difference did not reach statistical significance. Part of this quantitative discrepancy could be attributed to differences in animal gender between Gaeta et al. [14] and our study, because the effect of drugs varies with gender. In addition, we worked with younger animals (aged nine weeks when verapamil was administered), while Gaeta et al. [14] used mice aged 16 and 48 weeks.

On average, no treatment induced unsafe blood glucose levels (limit 63–176 mg/dl). After two days with a ketogenic diet, BGLs decreased as expected compared with baseline (Table 2). Although Tupper et al. [23] did not observe changes in BGLs in C57BL/6 mice after 12 hours on a ketogenic diet, 129 mice showed reduced BGLs (74±20 mg/dl) similar to that found in the present study after 48 hours (75.7±25.7 mg/dl).

The hypothesis of Experiment B was that suppression of myocardial uptake of ^18^F-FDG would enhance the visualization of nearby lesions, thus improving the diagnosis of several conditions such as infective endocarditis, coronary atherosclerotic lesions, and some metastatic lesions. Therefore, we tested the best protocol from Experiment A in a lung inflammation model generated in animals by tracheal administration of LPS. Visualization improved after two days on a ketogenic diet; according to the results of our previous experiments, this was the best approach to reducing myocardial uptake of ^18^F-FDG. PET images after administration of LPS showed that hot areas near the heart were more easily identified (Fig. 4D) when myocardial uptake was completely suppressed. Conversely, in animals in which myocardial uptake was not or only partially suppressed, it was much harder to distinguish inflammation from myocardial spillover (Fig. 4B–C). Lesion/myocardium ratio also improved in the animals with complete suppression compared with partial or null suppression, due to a lower background near the lesions (the heart). Although adherence is not, in theory, an issue in animal studies, palatability could affect animal food consumption. In addition, it is possible that the marked distress observed in the LPS animals changed their food consumption. Severe pulmonary distress could also have led to an increase in cardiac metabolic needs, forcing a switch to glucose as the metabolic substrate. Moreover, pulmonary distress may also lead to myocardial hypoxia. When the oxygen supply decreases, the heart protects itself by switching energy production to glycolysis [25], resulting in increased ^18^F-FDG uptake, as reported by McNulty et al. [26]. This could explain why the ketogenic diet was less effective in the LPS-challenged mice than in healthy animals. Therefore, the LPS model was not optimal for demonstrating the efficacy of the diet in reducing myocardial uptake of ^18^F-FDG. Nevertheless, the efficacy of the diet was well demonstrated in healthy animals. The LPS model was solely intended to illustrate better visualization of pulmonary lesions when myocardial uptake is suppressed. Our study provides the first validation of this methodology in animal studies, although previous clinical studies have examined the usefulness of high-fat protocols to visualize inflammation in coronary arteries [9] or metastases [5].

Several studies have evaluated the effect of high-fat diets on inflammatory diseases, such as atherosclerosis. In rodents, inflammatory gene expression is modified after six to ten weeks on a high-fat diet [27], [28]. Erridge et al. [29] reported an increase in circulating plasma endotoxin after a high-fat meal in healthy subjects; this endotoxin may be associated with the risk of atherosclerosis. As for ^18^F-FDG uptake, some authors have shown that hypercholesterolemia and age (which is equivalent to the duration of cholesterol loading) were consistently correlated with ^18^F-FDG uptake in large arteries in patients [30] and in the mouse aorta [31]. Thus, although we did not analyze the effects of ketogenic diet on ^18^F-FDG uptake in inflamed tissues, Williams and Kolodny [5] reported no differences in uptake between low-carbohydrate, high-fat diets and fasting (p = 0.30; SUV_range_ 1.8–5.6) in patients with chronic inflammation (osteoarthritis, tendinitis, dental). We cannot rule out the possibility that a high-fat diet can exacerbate lung inflammation and thus induce an increase in ^18^F-FDG uptake that further improves the signal-to-noise ratio. Notwithstanding, in order to better visualize this kind of lesion near the heart, it is still necessary to reduce myocardial uptake of ^18^F-FDG.

Interpretation of the data in this study is limited by the fact that the animal experiments were not randomized. Thus, treatments were always tested following the same order (baseline, verapamil, ketogenic diet, and sunflower seeds for experiment A). Although the optimal withdrawal period between treatments is unknown, fatty acid metabolism is suppressed when insulin levels increase after a regular meal [32], and the mean elimination half-life in single-dose administration of verapamil ranges from three to seven hours [33]. For these reasons, we thought a delay of four days between treatments would be more than sufficient, although there is no complete guarantee that this is so. Even though we know that myocardial uptake can sometimes be reduced by fasting, we did not follow this approach, because in our experience and in the experience of other researchers, it is often not effective [5], [8], [23]. This is also the main reason why we decided not to include a control group when we evaluated the ketogenic diet in the lung inflammation study (Experiment B). We did not measure food intake per animal, and this prevented us from studying the relationship between total dietary consumption and myocardial uptake of ^18^F-FDG. We also did not measure ketone bodies in the blood stream, which according to some authors might affect ^18^F-FDG uptake [24], [34], [35]. We were also not able to comment on cardiac glucose metabolism, because we did not measure the necessary specific parameters such as levels of glucose, insulin, and lactate or oxygen consumption. A final limitation was the relatively high dose of LPS (5 mg/kg), which, as mentioned above, produced severe pulmonary distress. This may have reduced the efficiency of ketogenic diet in the LPS mice compared to the healthy mice. A lower LPS dose might have maintained the efficacy of ketogenic diet.

In conclusion, these results show that two days on high-fat diets prior to ^18^F-FDG-PET imaging suppresses myocardial uptake in mice better than verapamil. Although a sunflower seed diet was slightly less effective than a ketogenic diet, it is much more affordable, thus making it a potentially good alternative in some situations. Our findings also demonstrate that a ketogenic diet improves visualization of inflammatory lesions near the heart.
